# Auditing YouTube’s recommendation system for ideologically congenial, extreme, and problematic recommendations

**DOI:** 10.1073/pnas.2213020120

**Published:** 2023-12-05

**Authors:** Muhammad Haroon, Magdalena Wojcieszak, Anshuman Chhabra, Xin Liu, Prasant Mohapatra, Zubair Shafiq

**Affiliations:** ^a^Department of Computer Science, University of California-Davis, Davis, CA 95616; ^b^Department of Communication, University of California-Davis, Davis, CA 95616

**Keywords:** YouTube, congeniality, recommendation algorithms, extremity, ideology

## Abstract

Algorithms of social media platforms are often criticized for recommending ideologically congenial and radical content to their users. Despite these concerns, evidence on such filter bubbles and rabbit holes of radicalization is inconclusive. We conduct an audit of the platform using 100,000 sock puppets that allow us to systematically and at scale isolate the influence of the algorithm in recommendations. We test 1) whether recommended videos are congenial with regard to users’ ideology, especially deeper in the watch trail and whether 2) recommendations deeper in the trail become progressively more extreme and come from problematic channels. We find that YouTube’s algorithm recommends congenial content to its partisan users, although some moderate and cross-cutting exposure is possible and that congenial recommendations increase deeper in the trail for right-leaning users. We do not find meaningful increases in ideological extremity of recommendations deeper in the trail, yet we show that a growing proportion of recommendations comes from channels categorized as problematic (e.g., “IDW,” “Alt-right,” “Conspiracy,” and “QAnon”), with this increase being most pronounced among the very-right users. Although the proportion of these problematic recommendations is low (max of 2.5%), they are still encountered by over 36.1% of users and up to 40% in the case of very-right users.

## Significance

YouTube’s algorithm is often accused of putting users in filter bubbles and generating rabbit holes of radicalization. However, evidence on these issues is inconclusive. We conduct a systematic audit of the platform using 100,000 sock puppets that allow us to isolate the influence of the algorithm in recommendations to ideologically congenial and increasingly extreme and problematic videos. YouTube’s algorithm recommends ideologically congenial content to partisan users, and congenial recommendations increase deeper in the recommendation trail for right-leaning users. Although we do not find meaningful increases in ideological extremity of recommendations, we show that a growing proportion of recommendations deeper in the recommendation trail come from extremist, conspiratorial, and otherwise problematic channels. This increase is most pronounced among the right-leaning users.

American society is deeply divided—there is a growing gap between the left and the right on key policies (1), hostility between partisans is increasing (2), and support for political violence and rejection of democratic norms are not uncommon (3). Although many factors contribute to the growing polarization and radicalization, the rise of social media has come under increased scrutiny. Critics observe that social media platforms place users in unique environments characterized by self-curated information flows, filtered through one’s social network, and reinforced by recommendation algorithms (4). This may lead to a feedback loop—a potential cycle of reinforcement. The worry is that this loop effect is prevalent on platforms and a major contributing factor to polarization.

Compared to individual biases and social homophily, algorithms are the least well-understood factor that profoundly influences online exposure. Recommendation algorithms are designed to optimize user engagement (5) by personalizing recommendations based on users’ past exposures and content viewed or shared by other similar users. Although this allows the user to see personally relevant content, problems arise in the case of politics (for a review, see ref. (6)). Then, such algorithmic personalization may lead to recommendations to politically congenial information, minimize exposure to diverse and dissimilar viewpoints, and potentially direct users to problematic contents. In extreme cases, these patterns could result in user radicalization or civil unrest (7, 8).

In this context, YouTube in particular is receiving increasing scrutiny from scholars, media, and regulators. It is the most popular platform, used by 81% of the US population and with a steadily growing user base (9). Over 70% of content watched on YouTube is recommended by its algorithm (10), which is proprietary and opaque to users and regulators. There are concerns that the algorithm exposes users to divisive, conspiratorial, and otherwise problematic content (11). Accordingly, YouTube has been described as a “long-term addiction machine” (8), accused of putting users in “filter bubbles” and “rabbit holes” and claimed to be “one of the most powerful radicalizing instruments of the 21st century” (12).

Despite the immense potential impact of YouTube’s recommendation algorithm, evidence to support these claims is inconclusive. First, does the algorithm indeed promote exposure to ideologically congenial videos? Second, does the algorithm drive users to increasingly extreme and radical political content? Whereas some studies reportthat YouTube tends to recommend like-minded and problematic videos (13–16), others provide evidence to the contrary (17, 18). For instance, Papadamou et al. (16) find that users have a 6.3% chance of encountering problematic recommendations when starting from moderate content. Similarly, Ribeiro et al. (17) show that many users migrate from ideologically moderate to extreme channels. In contrast, examining online behavioral traces of YouTube users, Hosseinmardi et al. (19) find little evidence that YouTube’s recommendation algorithm is driving attention to radical content. These conflicting conclusions are due to subtle but crucial differences in the methodologies. Some work relies on active measurements using untrained sock puppets (i.e., without any watch history) (17, 18) and thus cannot capture recommendation processes among actual users. In turn, the studies that rely on real user watch activity cannot tease apart the role of algorithmic recommendations from the actions of the user (17, 19, 20). More recent works on trained sock puppets find moderate filter bubbles (21). In *SI Appendix*, A, we review these prior works in more detail.

We address these gaps. We design a sock puppet–based auditing methodology that allows us to systematically and at scale isolate the influence of the algorithm in recommendations to ideologically congenial and increasingly extreme and problematic content. Trained sock puppets achieve a middle ground between untrained sock puppets and real users and can reconcile conflicting evidence in prior literature. We trained 100,000 sock puppets, watching a total of 9,930,110 YouTube videos from 120,073 channels, to reflect different ideology categories from very-left to very-right.

Our large-scale audit examines three key questions: Are recommended videos congenial with users’ ideology, especially deeper in the watch trail? Are recommendations deeper in the trail progressively more extreme, based on our classification of the ideological slant of each video? And, Do recommendations deeper in the trail direct users to conspiratorial, misinformative, or radical contents, based on extensive validated lists of problematic YouTube channels (17–19)?

We find that YouTube’s algorithm does recommend primarily congenial videos to its partisan users without, however, shielding them away from diverse and ideologically cross-cutting content. This congeniality is most pronounced in homepage recommendations and increases deeper in the watch trail for the very-right users. Although the videos recommended deeper in the watch trial do become significantly more extreme, these increases are substantively small. We also show that a growing proportion of recommendations deeper in the trail comes from YouTube channels categorized as problematic (e.g., “IDW,” “Alt-right,” “Alt-lite,” “Conspiracy,” “QAnon,” “WhiteIdentitarian,” etc.), and this increase is most pronounced among the very-right users (hence, less unlikely for the average YouTube user). Although the proportion of these problematic recommendations is rather low (max. 2.5%), they are still encountered by over 32% of users and up to 40% in the case of very-right users.

## Materials and Methods

### Overview of Audit.

In order to measure the extent to which YouTube’s recommendations are ideologically congenial with regard to the user, we conduct a systematic audit of the platform using “sock puppets”: automated browser instances that mimic YouTube users by watching videos and gathering recommendations. The audit consists of two main phases: training and then testing sock puppets. The sock puppets are trained by watching videos of a particular political ideology and then their personalized recommendations are tested in a controlled manner. We test two types of YouTube recommendations: 1) those on the homepage and 2) up-next recommendation trails starting from a seed video.^*^

#### Estimating video ideology.

We estimate the ideological slant of a YouTube video by analyzing its audience on Twitter (22). We emphasize that our approach substantially extends prior work, which analyzes political ideology at a coarse channel-level granularity (17–19), by estimating slant at the finer granularity of individual videos. Because not all videos posted by a channel reflect that channel’s ideology, it is erroneous to assume that each video shares the ideology of a channel (see ref. (23) for evidence on news domains versus individual news articles).^†^ In addition, established ideological categorizations exist for only a handful of YouTube channels, and so, it is not possible to capture the slant of individual videos from thousands of niche and less popular channels existing on YouTube.

To estimate the video-level slant of any given YouTube video, we collect the tweets mentioning the video using the Twitter API and check if the authors of those tweets follow a set of well-recognized partisan elites, i.e., landmarks, whose political ideology is clearly established as either left (i.e., liberal) or right (i.e., conservative) (24). To minimize the threat that the estimates are unstable or biased by a particular tweet, we only estimate the slant if the number of landmarks is greater than 12. The landmarks followed by a user provide insight into the user’s political ideology, as Twitter users are more likely to follow other users of the same ideology, as consistent with the classic homophily assumption and the spatial models of ideology that rely on this assumption (22). For the tweets mentioning a given YouTube video, we count the total number of very-left (L) and very-right (R) landmarks followed by the authors of those tweets and estimate the slant on a scale ranging from −1 (very-left) to +1 (very-right) as

[1] $$Slant=\frac{R-L}{R+L}.$$

In addition to their continuous slant estimate, we also categorize videos into the following slant ranges [−1,−0.6), [−0.6,−0.2), [−0.2,+0.2), [+0.2,+0.6), [+0.6,+1] as very-left, *left*, *center*, *right*, and very-right, respectively. These categories correspond to the sock puppet ideologies we specify during training. In *SI Appendix*, B, we offer additional details on the methodology, outline the robustness of our approach, present evidence for the benefits of video-level estimation, provide more details regarding the thresholds, and validate the channel- as well as video-level slant estimations against human-based methods.

#### Training sock puppets.

We train sock puppets in the following five ideologies: very-left, *left*, *center*, *right*, and very-right. Combined, we train over 100,000 sock puppets and each watches 100 randomly sampled videos from its assigned ideology for 30 s each, in accordance with the insight from ref. (15).^‡^ The videos in each ideology are collected from known politicians, news outlets, pundits, and journalists, and their ideology is estimated using the approach discussed earlier. In *SI Appendix*, C, we offer additional details on the training, the videos used, and their source (*SI Appendix*, C.1) and on the ecological validity of the approach vis-a-vis YouTube watch histories of human users (*SI Appendix*, C.2).

#### Testing sock puppets.

Once a sock puppet has been trained, we first collect the video recommendations on its YouTube homepage. The homepage is expected to contain recommendations of interest to the sock puppet based on their watch history.^§^

Second, we gather the up-next video recommendations from a YouTube video randomly selected from all the training videos (henceforth, referred to as the seed video) and build a recommendation trail, mimicking YouTube’s autoplay functionality. Because the sock puppet follows the recommendations, this trail can show whether recommendations are increasingly more congenial and also whether following the recommendations leads users to more extreme and radical content deeper in the trail.

Table 1 provides a summary of the scale of the data collected through the sock puppets. From the collected recommendations, we test whether i) the homepage recommendations are ideologically congenial with regard to the user; and also whether following the up-next recommendations ii) increases exposure to ideologically congenial content; iii) leads to more ideologically extreme content, and iv) directs users to a growing number of channels that are conspiratorial, radical, or otherwise problematic.

**Table 1.** Data collection statistics for 100,000 sock puppets

	Training	Testing	Total
# of videos watched	9,930,110	5,393,820	15,323,930
# of unique videos	23,735	381,153	399,935
# of channels	1,256	119,811	120,073

## Results

### Ideological Congeniality in Recommendations.

We start by examining congeniality of recommendations in both the homepage and the up-next recommendations. Specifically, we test whether the videos recommended to the sock puppets by YouTube’s algorithm are consistent with the sock puppets’ ideology as determined by the training, e.g., are (very) left sock puppets recommended (very) left videos and are (very) right sock puppets recommended (very) right videos.

### Homepage Recommendations.

Homepage recommendations are shown to the user when they first visit YouTube and are organized in a grid that spans two rows and typically four columns. This implies that the user is likely to see eight recommendations when they first visit YouTube. We thus focus on the first recommendation (top 1, n=1), presumed to be most interesting and important to the user, and all the recommendation a user sees on their homepage without scrolling down (top 8, n=8), analyzing whether these are consistent with the user’s ideology.

**Fig. 1.**
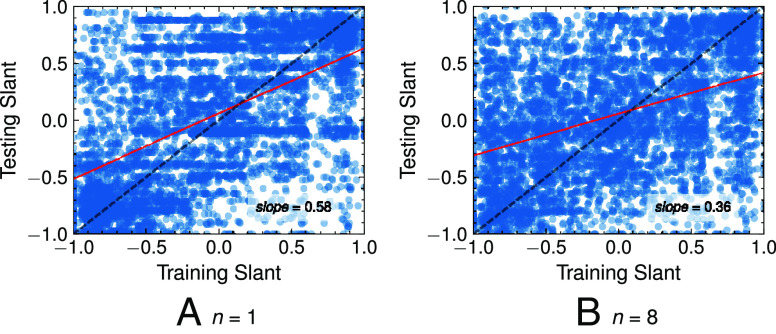
Ideological congeniality between slant of training videos and the slant of top-1 (*n*= 1) and top-8 (*n*= 8) homepage recommendations. The higher the slope of the red line, the more congenial the recommendations to the training.

We first investigate the impact of sock puppet training on the slant of the homepage videos recommended during testing. Fig. 1*A* and *B* report the congeniality between the slant of individual videos watched in training and videos recommended in testing. We quantify the congeniality in terms of the slope of linear regression (shown by the red line in the figure). A slope of 1.0 (i.e., the diagonal) would indicate that recommendations are perfectly congenial (e.g., 100% very left/right videos recommended to a very left/right sock puppet). On the other hand, a slope of 0.0 (i.e., a horizontal line in the middle) would indicate that the recommendations are truly diverse (e.g., 50% left and 50% right videos) or perfectly neutral. (e.g., 100% of videos classified as ideologically moderate). A slope >0.0 would indicate congeniality in the recommendations and suggest that the training impacts the slant of the videos recommended during testing.

Fig. 1*A* and *B* show that the slopes are 0.58 and 0.36 for n=1 and n=8, respectively. This suggests that the homepage recommendations are congenial albeit less congenial than the training videos, which—again were exclusively from within one specific ideological category. The fact that YouTube’s algorithm does not exclusively recommend 100% congenial videos is not surprising because the recommender system is designed to recommend “a mixture of personalized recommendations, subscriptions, and the latest news and information” (27–29). The exact distribution of recommendations is not disclosed, but it is reasonable to assume that the noise introduced by these “trending” or “popular” videos recommended to all users would result in recommendations that are not 100% consistent with the training of our sock puppets.

**Fig. 2.**
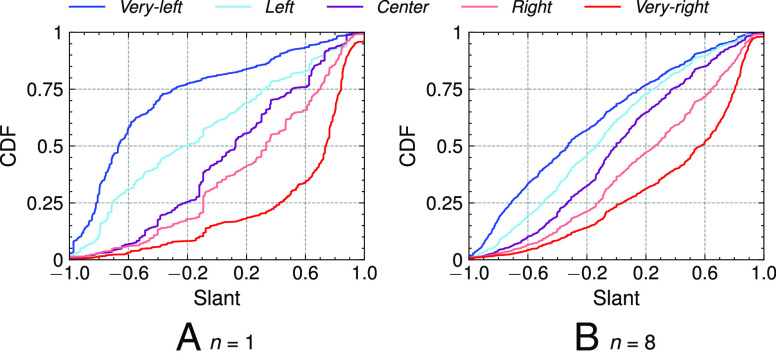
Probability (cumulative distribution) of encountering a video of certain slant in the top-n homepage recommendations. For the very-right sock puppet, there’s a 65.5% chance that the first homepage recommendation is congenial (i.e., has a slant ≥+0.6).

We next compare the congeniality across sock puppets of different ideologies. Fig. 2*A* and *B* report the probability that the top-1 and top-8 recommendations on the homepage are congenial. Fig. 2*A* shows that for the very-left sock puppets, the probability that the first recommendation is also very-left is 58.0%. Similarly, the probability that very-right sock puppets receive congenial top-1 recommendation is about 65.5%. This figure further shows that the *center* sock puppet sees 30.1% of congenial (i.e., moderate) recommendations but also a high percentage of *right* (20.3%) and very-right (24.1%) recommendations.

Going beyond the top-featured recommendation, Fig. 2*B* plots the probabilities for the top-8 recommendations. This represents all homepage recommendations a user would see when they first visit YouTube. The probabilities of congenial recommendations are similar albeit slightly lower relative to just the top-1 (33.6% for a very-left sock puppet; 48.0% for the very-right sock puppet).

The differences between the distributions shown in both figures are statistically significant (P<0.05). Two-sided pairwise Kolmogorov–Smirnov tests between the *center* sock puppet and each of the other sock puppets show that the ideological slant of homepage videos recommended for very-left, *left*, *right*, and very-right sock puppets is different from that of *center* sock puppets as can be seen by the statistic values in *SI Appendix*, Table S3 in D.^¶^

These test statistics further indicate that the magnitude of the differences is larger for very-right sock puppets as compared to very-left sock puppets. Specifically, for n=8, the recommendations for very-right sock puppet are significantly more right-leaning compared to the *center* recommendations than those for very-left sock puppet.^#^

**Fig. 3.**
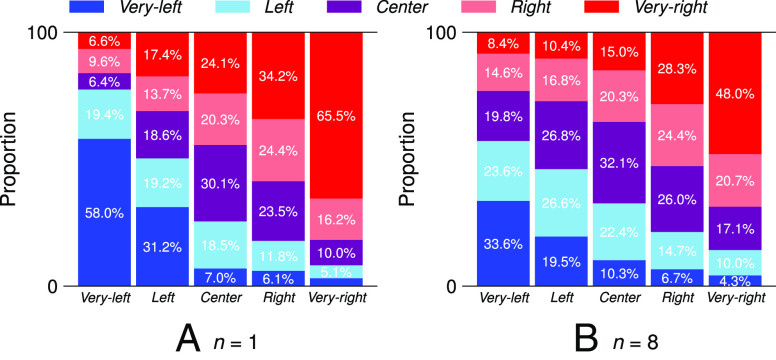
The distribution of ideological categories in top-1 (*n*= 1) and top-8 (*n*= 8) homepage recommendations. For the very-left sock puppet, the top-1 recommendation is 58% likely to be congenial.

To offer additional nuance, we plot recommendations to the other slant categories. After all, for instance, recommendations to left-leaning videos are also congenial for very-left users. Fig. 3*A* and *B* show the proportions of the slant categories in top-1 and top-8 homepage recommendations for each of the five sock puppet ideologies.

Extending the above evidence for congeniality, we find that for the very-left sock puppets, a total of 77% of the top-1 recommendations are very left and left. This proportion is yet higher for the very-right sock-puppets, for whom 81.7% of recommendations are very right and right. These proportions slightly decrease when looking at top-8 homepage recommendations, yet still remain the majority (57.2% and 68.7%, respectively).

Despite this dominant congeniality, the algorithm also recommends ideologically diverse and even cross-cutting content. For instance, the very-left sock puppets encounter very right (6.6%) and right (9.6%) content in the top-1 recommendations (and 8.4% and 14.6% in the top-8 recommendations). These cross-cutting recommendations are less frequent for very-right sock-puppets: Those have a 3.2% chance of seeing very left and a 5.1% chance of seeing left videos in top-1 recommendations (4.3% and 10%, respectively, in the top-8 recommendations). In general, however, we see an ascending pattern in the percentage of very right and right recommendations as we go left-to-right from the very-left to the very-right sock puppet. A similar pattern—indicative of congeniality—can be observed for very left and left recommendations as we go right-to-left.

### Up-Next Recommendations.

Do congenial recommendations increase the longer users watch YouTube videos? We now analyze whether following the up-next recommendation trail promotes exposure to a growing number of ideologically congenial content. To measure the degree of this exposure, we define the following exposure metric.

[2] $$E_{d}^{\;\text{ideology}}=\frac{\%\;\;\text{of}\;\;{\text{videos}}_{d}^{\;\text{ideology}}}{\%\;\;\text{of}\;\;{\text{videos}}_{0}^{\;\text{ideology}}}.$$

This metric measures the increase in the percentage of videos of a given ideology at depth d over depth 0 (start of the trail). For parsimony, we check for values of ideology ∈ {very-left, very-right} for d up to 20 videos.

**Fig. 4.**
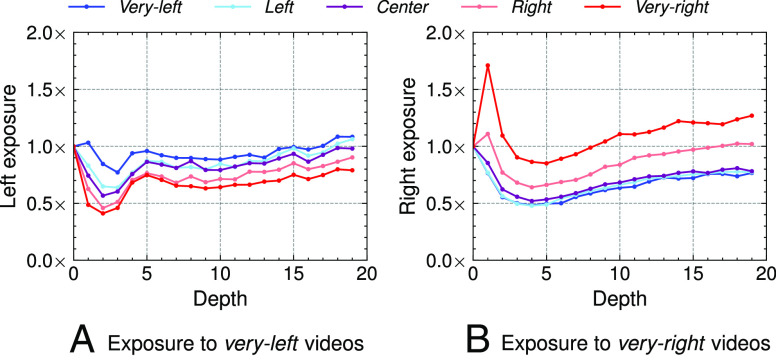
Exposure to ideologically very-left and very-right videos in the up-next recommendations. The colored lines correspond to the ideology of the sock puppet. The x-axis corresponds to the depth d and the y-axis to the exposure E for the very-left and very-right ideologies. For example, in the right-hand figure at depth 1, the very-right sock puppet is exposed to 1.7× more very-right videos.

Fig. 4 shows the values of E for both ideology ∈ {very-left, very-right} and as d varies from 0 to 20. There is an increase in the number of very-right recommendations as the very-right sock puppet follows the trail. The group of very-right sock puppets sees the highest values of very-right exposures at depth 1 (1.7×), and 1.3× at depth 20. In other words, there is a 37% increase in very-right recommendations for the very-right sock puppets at the end of the trail, with the overall pattern pointing to a further increase. In contrast, the very-left sock puppets initially experience a drop in very-left recommendations, a 5% increase at depth 1, and an overall stability in the number of ideologically congenial recommendations deeper in the trail.

In sum, following the recommendation trail increases the odds that right-leaning sock puppets will continue to watch ideologically congenial videos and does not increase recommendations to congenial videos for left-leaning sock puppets.

### Radicalization via Recommendations.

We now examine whether YouTube’s algorithm directs users to increasingly more extreme and politically problematic content. First, we rely on our estimated ideological video slant, which ranges from −1 to +1, to test whether recommendations deeper in the trail become more extreme (i.e., closer to −1 for the very-left and closer to +1 for the very-right sock puppets).^‖^ We do this for all five ideology categories. Second, we use the previously compiled lists of 4,150 politically problematic YouTube channels (17–19). We consider the following categorizations: “IDW,” “Alt-right,” “Alt-lite” from the list by Ribeiro et al. (17) and “AntiSJW,” “Conspiracy,” “MRA,” “ReligiousConservative,” “Socialist,” “QAnon,” and “WhiteIdentitarian” from the list by Ledwich et al. (18). *SI Appendix*, E presents the list of channel categories considered.

**Fig. 5.**
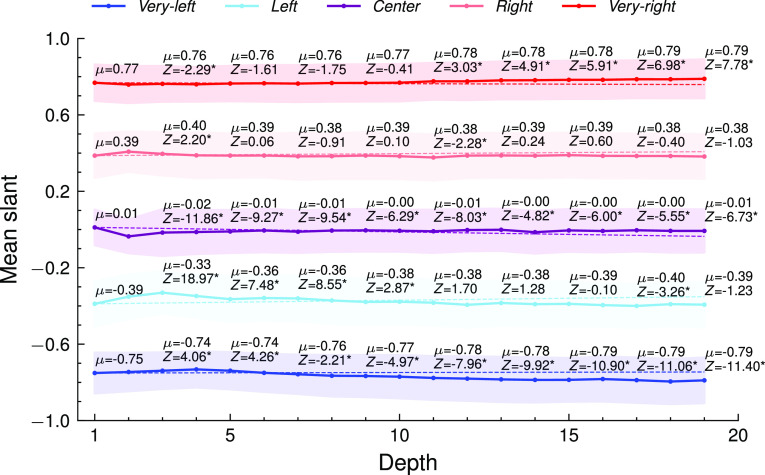
The mean slant of the ideologically congenial videos watched by each ideology category in the up-next recommendations. For example, the *Top* red line corresponds to the mean slant of the very-right videos watched by the very-right sock puppet. The dotted and dashed lines correspond to 1-SD and mean slant at depth 1 of the trail, respectively. The mean and SD become progressively extreme for the very-left and very-right sock puppets.

Addressing the potential increases in extreme recommendations, Fig. 5 plots the mean and SDs of the distributions at different depths. The mean slant of the videos for the very-left and the very-right sock puppets becomes only slightly more extreme as the sock puppets traverse the trail. For the very-left sock puppets, it increases from −0.75 to −0.79, and for the very-right ones, it increases from +0.77 to +0.79. The movement of the SDs also suggests that the recommendations become more extreme deeper in the trail for these groups. Although these changes are statistically significant, as reported by the Z-test statistic, they are small in magnitude. For more moderate sock puppets, those in the *left*, *center*, and *right* ideologies, this progression in extremity is not detected.

**Fig. 6.**
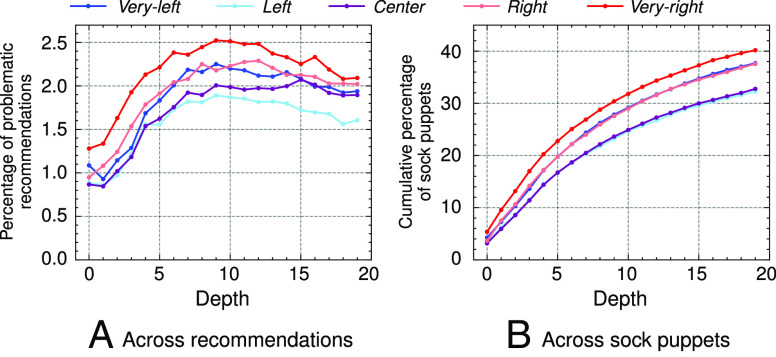
Percentages of problematic video recommendations across (*A*) all recommendations and (*B*) across all sock puppets. The figure on the *Left* shows the percentage of problematic recommendations over all recommendations at each depth. The figure on the *Right* shows the cumulative percentage of sock puppets that encountered one or more problematic recommendations up to that depth.

Addressing the recommendations to the problematic channels, Fig. 6*A* plots the percentage of up-next recommendations to all the radical, conspiratorial, or extremist channels from the lists. First, the proportion of problematic channels is rather small overall. Nevertheless, we observe increases in these recommendations deeper in the trail, from 1.2% at depth 1 to 2.5% at depth 10. Although this is not a dramatic increase, the number of recommendations to problematic channels that the sock puppets encounter deeper in the trail is far from trivial in absolute terms. Specifically, by following the up-next recommendations, the very-right sock puppets encounter the greatest number of problematic channels, 22,146, likely due to the majority of these channels being right-leaning, followed by the *right* sock puppets at 19,239. Close behind, the very-left sock puppet sees a total of 17,374 recommendations to problematic channels throughout their watch trail, the *left* sock puppet sees 15,132 such recommendations, and the *center* sock puppet sees 16,101.^**^

In addition, Fig. 6*B* plots the cumulative percentage of sock puppets that encounter problematic recommendations at each depth. We see that this percentage steadily increases at the start before plateauing around depth 10. On average, 36.1% of the sock puppets encounter recommendations to problematic channels in the up-next trail. This number varies between 40% for the very-right sock puppet and 32% for the *left* and *center* sock puppets.

Again, although the absolute majority of recommendations are not to problematic content, these patterns suggest that the various radical, conspiratorial, or extremist channels are in fact recommended more frequently the longer the user engages with the platform. Because these channels were not used in the sock puppet training, these organic recommendations to problematic content not previously seen are disturbing.

Last, we note that the prevalence of problematic recommendations decreases toward the end of the trail, with the decline starting at depth 10 (even though these problematic recommendations are still substantially higher than they were at depth 1). This is likely due to the fact that the channels recommended deeper in the trail are not categorized or included in the original list compiled from refs. (17) and (18).^††^

### Additional Analyses.

We additionally examined the effects of two alternative ways to train sock puppets on recommendations: 1) single-channel-trained sock puppets and 2) truly ideologically heterogeneous sock puppets. First, we trained over thirty sock puppets for each, MSNBC and Fox News. We find pronounced ideological congeniality in the recommendations, i.e., the top-1 recommendation is almost 100% congenial to the ideology of the news channel used in training (left for MSNBC and right for Fox News). The results also suggest that only the political leaning of both MSNBC and Fox News, not the channels themselves, were reflected in the recommendations. Specifically, of the 184 homepage recommendations collected from the MSNBC sock puppet, only 23 were from MSNBC itself. Similarly, of the 176 recommendations collected from the Fox News sock puppet, only 21 were from Fox News itself. Furthermore, a growing proportion of these recommendations also came from problematic channels, indicating that potentially radical or conspiratorial channels also appear in the recommendations despite the fact that the sock puppets were only trained on rather mainstream news channels. These results and corresponding figures are shown in *SI Appendix*, F.1.

Second, we trained over thirty truly ideologically heterogeneous sock puppets on 40% very-left, 20% *center*, and 40% very-right training videos. Examining recommendation congeniality at top-1 and top-8 homepage recommendations, we find that these recommendations are heavily right-slanted despite having a balanced composition of left and right content during training. This suggests that the recommendation system is more heavily influenced by very-right content. Again, the truly heterogeneous sock puppets also see an increasing proportion of problematic recommendations in the up-next trail. *SI Appendix*, F.2 presents the details and the relevant figures.

## Discussion

Algorithmic recommendation systems of social media platforms are solely designed to optimize user engagement. Showing people more of what they like is a feature of all recommendation systems and is not on its own alarming. Nevertheless, in the case of political content, there are concerns that personalization and the incentive to maximize user engagement lead to a situation in which the recommended content amplifies users’ prior biases and minimizes exposure to contrasting viewpoints (6).^‡‡^ In extreme cases, personalization and optimizing engagement may direct some users to increasingly more radical, conspiratorial, or otherwise problematic content.

We offer a holistic understanding of whether and how the algorithmic recommendation system of YouTube drives exposure to ideologically congenial and progressively more extreme and problematic videos. First, we find that the recommended content is closely aligned with prior political leanings of partisan users, represented by our trained sock puppets. The majority of videos recommended as the top recommendation and a large part of all the videos appearing on the users’ YouTube homepage are ideologically like-minded. This is not to say that YouTube’s recommender system shields the users from diverse content, as can be expected given that the system is designed to also recommend trending videos and the latest information. For instance, we find that the testing videos are less congenial than the exclusively congenial videos on which the sock puppets were trained. Similarly, the users’ homepages feature recommendations to moderate and cross-cutting content: even the very-left and the very-right users may see videos that are diverse or of opposing political ideology (see also refs. (17–19)). Again, however, ideologically congenial recommendations dominate, especially for the very-right users.

Second, it is not the case that congeniality linearly increases as the users traverse the up-next recommendation trail. This finding aligns with a recent audit, which also shows that personalization is more pronounced on the homepage than in up-next recommendations (21). In our audit, the very-left sock puppets do not encounter a growing number of very-left videos as they follow the up-next recommendations. Nevertheless, for the very-right sock puppets, their chances of encountering very-right recommendations increase by 37% as these sock puppets continue engaging with YouTube. This increase is far from trivial and further supports the finding of greater congeniality for this ideological group.

Third, the videos recommended to very-left and very-right sock puppets do become significantly more extreme the longer these sock puppets engage with YouTube. These shifts, however, are not substantively large and unlikely to be democratically impactful. For instance, the recommendations for the very-left sock puppets move from −0.75 to −0.79 between the start of the watch trail and its end (depth 20), and those for the very-right sock puppets move from +0.77 to +0.79 on an ideology scale from −1 to +1.

Ideological extremity aside, following the trail of YouTube’s up-next recommendations leads our sock puppets, partisan and moderate alike, to previously unseen problematic YouTube channels such as “IDW,” “Alt-right,” “Conspiracy,” “Socialist,” “QAnon,” or “WhiteIdentitarian,” among others. The very-right and *right* sock puppets are most likely to encounter them in their watch trail, yet even the moderate sock puppets are recommended a growing number of problematic channels as they follow up-next recommendations (see *SI Appendix*, F.2 for similar evidence for truly heterogeneous sock puppets, and *SI Appendix*, F.1 for those only trained on MSNBC or Fox News videos). Consistent with past work (19, 20), recommendations to these channels represent a fraction of all the recommendations the sock puppets receive, never surpassing 2.5% on average. Similarly, the increases in these recommendations are not steep, with the maximum increase being 1.3% on average. Yet, because these problematic channels were not included in the training, their appearance in the up-next recommendation trails for over 32% of sock puppets is problematic. Furthermore, because 70% of YouTube’s content is watched from recommendations (10) without requiring any additional actions or decisions from the users, this pattern of recommendations may activate the cycle of radicalizing exposures.

In short, although it is unlikely that an average YouTube user will be steered to more extreme content, the user may encounter videos that promote conspiracy or misinformation, among other problematic messages deeper in the recommendation trail. The finding that this is most likely for far-right users is consistent with Chen et al. (20) who show that “many racially resentful people are not only watching large numbers of videos from alternative or extremist channels, but also are shown recommendations for more such videos when they do so, further increasing exposure to potentially harmful content.” Undeniably, the most pernicious potential effects of such recommendations and exposures would emerge among the already predisposed and susceptible users (20).

Fourth, the audit suggests the presence of right-wing bias in YouTube’s algorithm. The very-right sock puppets encounter more congenial recommendations than any other ideological group, are the only group directed to a growing number of congenial far-right recommendations deeper in the trail, and also are recommended disproportionately more problematic channels than the other groups. We additionally find that the moderate sock puppets encounter more right-leaning than moderate or left-leaning content and that the heterogeneous sock puppets and those trained on one single news source are also directed to more right- than left-leaning content (*SI Appendix*, F.1). This finding aligns with recent accusations that some platforms may promote right-leaning content (34) and survey-based evidence that Republicans are twice as likely as Democrats to see right-leaning videos on YouTube (35).

We acknowledge that we do not have data on what real users click on and how they react to algorithmic recommendations. Our sock puppets always followed up-next recommendations and so our audit—as other similar audits (16)—shows the effects that would emerge if users followed the recommendations. Future audits should attempt to account for how social media users interact with recommendations, and how individual and algorithmic factors together drive potential filter bubbles or algorithmic radicalization on platforms (36).

We also acknowledge that, naturally, we could not analyze attitudinal or behavioral outcomes of the tested recommendations. Media messages do have effects on individual cognitions, opinions, and actions, and these effects are cumulative (37). Therefore, over time, exposures to the videos in our audits could influence users. Studies extending our work to actual YouTube users and testing over time effects of algorithmic systems are needed.

Furthermore, our audit was not designed to address questions such as how much does the algorithm change over time, whether the results change if a user watches more or less of a video, or what happens if a user stops watching and then returns to a video. Our objective was more foundational: to isolate the impact of the users’ ideological leaning on algorithmic recommendations, not to isolate the different workings of the algorithm. We can shed light on some of these parameters, however. In *SI Appendix*, F, we show that training sock-puppets on videos from only one news source or training sock-puppets on a truly ideologically heterogeneous mix of videos leads to very similar effects. In addition, we advance https://youtubeaudit.com, a tool built as a result of this project where we automatically publish daily recommendations for the left, moderate, and right sock puppets to longitudinally demonstrate how changes in the algorithm influence recommendations, also in response to some key political events.

We also acknowledge that YouTube (and other platforms) does not function in isolation from an overarching information and communication ecology of its users. For instance, those who consume extreme or problematic videos on YouTube are likely to consume similar contents elsewhere (19, 38). Such off-platform consumption may drive radicalization as well. In a related vein, in addition to the algorithm, there are other exogenous and endogenous factors that reciprocally influence one another in driving ideologically congenial, extreme, or problematic exposures. For instance, according to a “supply and demand” framework (39), YouTube only serves as a medium that allows extremist content publishers (supply) to reach an already radicalized community of users (demand). YouTube offers incentives to content publishers to post problematic content (e.g., monetization), which drives up this supply. Thus, user radicalization on YouTube is driven by this interplay of supply and demand for extreme content, in which recommendation algorithm also plays a role.

In sum, our audit reveals the prevalence of congenial recommendations, limited increases in ideological extremity, and growth in recommendations to highly problematic channels on YouTube. To better understand these complex findings, researchers should continue identifying other factors that influence exposures to congenial, extreme, or problematic contents on YouTube (19, 39) and platforms should make their recommendation systems more transparent to users, scholars, and regulators. Considering the current political climate in the United States, greater transparency in social recommendation systems and also auditing these systems for filter bubbles and rabbit holes of radicalization is timely and needed.

## Supplementary Material

Appendix 01 (PDF)

## Data Availability

The sock puppet recommendations, their metadata, estimated slants, and list of landmarks have been deposited in the Open Science Framework (https://osf.io/gvsk5/) (40).

## Footnotes

^*^The research was submitted to the UC Davis Institutional Review Board (IRB). The research was determined to be exempt because it does not involve human subjects. The research was also reviewed by lawyers for the University of California who noted legal precedent for why this research does not violate the Computer Fraud and Abuse Act, YouTube’s terms of service, or other applicable laws—especially in light of important free speech and other First Amendment considerations.

^†^For comparison, we also provide our results using a channel-level slant estimation approach in *SI Appendix*, G.

^‡^For ads that appear during watch, we skip them if possible and wait until they are over if not.

^§^We note that these sock puppets are not signed into a specific Google account because that requires phone verification which is difficult to do so at scale for 100,000 sock puppets. However, there is evidence that recommendations are personalized even without signing in through the use of cookies (25). Although YouTube also uses Google account activity to personalize recommendations (26), because we are not signed-in, it is reasonable to assume that the YouTube watch history is the sole factor influencing recommendations.

^¶^We consider the *center* sock puppet as the baseline because of the closeness of its median to zero.

^#^In addition, there is a noticeable shift around 0.2 slant where the very-right distribution shifts farther right than the *right* distribution. In turn, the gap between the very-left and *left* distributions at the −0.6 slant, the cut-off between the two categories, indicates that the very-left received more left-biased recommendations than the *left* sock puppet. This gap decreases around as n→8 where the very-left sock puppet has recommendations closer in slant to the *left* sock puppet’s recommendations. In sum, the right-leaning sock puppets have a higher deviation from the *center* than the left-leaning sock puppets when more homepage recommendations.

^‖^For parsimony, we consider the distributions of videos corresponding to the sock puppets’ ideology. For instance, for the very-left sock puppets, we look at how the slant distributions for the very-left category videos vary as we traverse deeper into the recommendation trail.

^**^To generate these numbers, we add the recommendations to the problematic channels between depth 1 and 20 for each category of sock-puppets. The total number of recommendations collected from the very-left, *left*, *center*, *right*, and very-right sock puppets were 1,650,151, 1,634,174, 1,598,058, 1,560,922, and 1,535,263 respectively.

^††^Specifically, 37.9% of the channels at depth 1 appear in the entire list in refs. (18) and (17), problematic and nonproblematic included. At depth 20, this decreases to 21.6%, leading to the detected drop. This likely leads to an underestimation of the actual proportions of problematic channels recommended.

^‡‡^Exposure to a diverse array of content has beneficial effects on citizens’ attitudes, knowledge, understanding, and social cohesion (30). In contrast, exposure to like-minded perspectives reinforces attitudes, promotes out-party hostility, and has other negative consequences (31–33). Although what aggregate balance of exposure to neutral, congenial, or cross-cutting views is most beneficial remains an open normative question, there is general agreement that citizens need to be familiar with a range of viewpoints from across the political spectrum.
