# Supplementary material for: Auditing YouTube’s recommendation system for ideologically congenial, extreme, and problematic recommendations
Source: Proc Natl Acad Sci U S A. 2023 Dec 5;120(50):e2213020120. doi: 10.1073/pnas.2213020120 (PMC10723127; doi:10.1073/pnas.2213020120)
Supplement: Supplementary file 1 — Appendix 01 (PDF) [file pnas.2213020120.sapp.pdf]

PNAS
www.pnas.org

Supplementary Information for

Auditing YouTube's Recommendation System for Ideologically Congenial, Extreme, and Problematic Recommendations

Muhammad Haroon, Magdalena Wojcieszak, Anshuman Chhabra, Xin Liu, Prasant Mohapatra, Zubair Shafiq

Muhammad Haroon.
E-mail: mharoon@ucdavis.edu

This PDF file includes:

Supplementary text
Figs. S1 to S11
Tables S1 to S3
SI References

Supporting Information Text

A. Related Work. YouTube has been accused of putting its users in "rabbit holes" (1) and its algorithm has been described as a "long-term addiction machine" (2). This is a grave concern because over 70% of watched content on YouTube is via recommendations (3) and the top recommendation is typically played automatically after the currently-watched video. Thus, the fears are that, unknowingly and unwillingly, some audiences encounter ideologically consistent content (i.e., filter bubbles; (4)) and are at risk of being radicalized through the actions of the recommendation system (i.e., rabbit holes). The work on the existence of these algorithmic phenomena on YouTube is not fully conclusive, as we detail below.

A.1. Auditing YouTube's recommendation system. To systematically examine ideological congeniality and radicalization in algorithmic recommendations, there have been several audits conducted by the academic community. These report mixed evidence, largely due to methodological differences between these audits.

One line of research studies YouTube through active measurements using sock puppets. For instance, Ribeiro et al. (5) used untrained sock puppets (i.e., without any watch history) to show that about 5% algorithmic pathways led from ideologically moderate to extreme channels through video and channel recommendations. Similarly, Ledwich et al. (1) also relied on untrained sock puppets to show that 16% of the algorithmic pathways (11% moderate→left and 5% moderate→right) led from ideologically moderate to extreme channels through video recommendations. However, these studies assumed that YouTube's personalized recommendations for trained sock puppets are not different from those for untrained sock puppets (6, 7). This likely underestimates the prevalence of algorithmic filter bubbles and radicalization pathways.

Another line of research has used passive measurements of real user activity. An analysis of video comments by Ribeiro et al. (5) shows that a significant percentage of users migrate from ideologically moderate to more extreme channels on YouTube. Hosseinmardi et al. (8) longitudinally analyzed the browsing history of more than 300,000 users to understand user migration from moderate to extreme videos and vice-versa. Their analysis showed that a significant percentage of users migrated to non-news content and a slightly lower percentage did not migrate at all, i.e., users watching extreme news videos continued to do so. They also found that 8.5% and 31.8% of extreme video views originate from homepage and video recommendations, respectively. Furthermore, in a report for the Anti-Defamation League (ADL), Chen et al. (9) analyzed browsing history of 915 users to show that 9.2% of users watched a video from an extreme channel but less than 1% of the recommendations that users actually followed were from an extreme channel. This line of work is very important. However, by using real user's browsing histories, it cannot tease apart the role of algorithmic recommendations from the actions of the user.

On the one hand, audits analyzing sock puppets disregard the impact of sock puppet training, which is crucial for personalized recommendations and observing exposure to ideologically consistent or radical content on YouTube in practice. On the other hand, audits analyzing real user activity cannot tease apart the dependencies between algorithmic and other factors driving radicalization. Trained sock puppets achieve a middle ground between untrained sock puppets and real users for auditing purposes because (i) they trigger the feedback loop effect that real users (but not untrained sock puppets) experience and (ii) the observed radicalization can be attributed solely to YouTube's recommendations (which is not possible for studies of real users). In fact, recent work has adjusted this framework of trained sock puppets to audit misinformation in YouTube's recommendation system (7, 10–13). Extending their prior work, Ledwich et al. (14) used trained sock puppets to study personalized recommendations. Similarly to our results, they find moderate ideological congeniality for most personas, which is more pronounced on the homepage than in up-next recommendations.

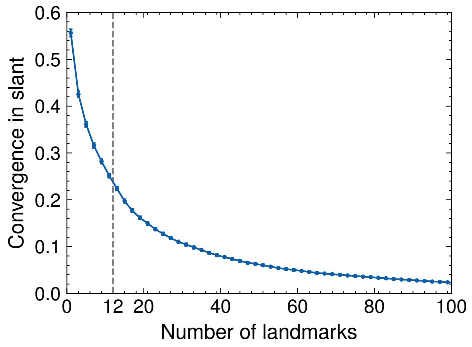

Fig. S1. Convergence of the estimated slant to its final value as the number of landmarks varies.

B. Estimation of the Ideological Slant of YouTube Videos.

B.1. Impact of number of tweets. We aimed to ascertain that our video-level ideology estimation is robust. For one, for a stronger signal, we only estimated the slant of a video if the number of partisan landmarks was greater than 12. In other words, we only consider the estimated slant of a video if at least 12 partisan landmarks (well-known political elites) were identified among the lists of the accounts followed by the users who tweeted the video. The list of landmarks comes from Le et al. (15). Restricting the popularity threshold minimizes the chances that the estimates are unstable or biased by any particular tweet alone.

We justify the choice of 12 landmarks by measuring how the slant varies if fewer landmarks are considered. Figure S1 shows how the estimated slant converges to its final value as we increase the number of landmarks for 10,000 randomly sampled videos in our dataset. At just one landmark, the slant differs from its final value by ±0.6. As the number of landmarks increases to 12 (the threshold we considered), this number drops to ±0.2. This number is consistent with the findings of Le et al. (15) who also considered a threshold of 12. Note that an increase above this threshold of 12 landmarks does not lead to substantial decrease in convergence toward the final value, and setting the threshold higher would lead us to discard a higher number of videos from our analysis. Furthermore, we highlight that the average number of landmarks per video in our dataset is 58 where the slant differs from its final value by ±0.049. Thus, we have a decent signal for computing the slant for a high number of videos in our dataset.

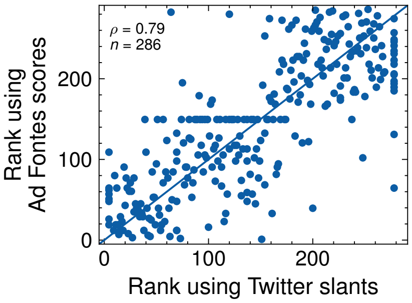

Fig. S2. Spearman's rank correlation (ρ) of 286 video articles from AdFontes and their estimated slant using the Twitter approach.

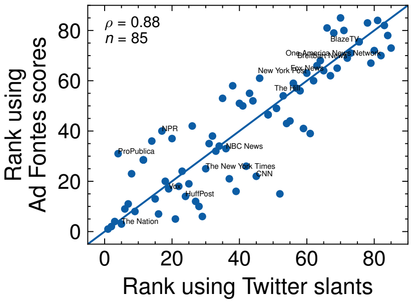

Fig. S3. Spearman's rank correlation (ρ) of 85 channel-level scores from AdFontes and their channel-level slant using the Twitter approach.

B.2. Validating the Approach. We assess the validity of our Twitter-based ideology slant estimation by comparing our scores with the Ad Fontes Media Bias Chart (16)*. Ad Fontes' scores are based on manual labeling of articles, podcasts, and videos, with each individual article and episode rated by at least three human analysts from across the political spectrum (right, left, and center, based on self-reports). The scores are assigned along two dimensions: ideological bias and reliability, of which only the former is of relevance to our validation. In addition to article-level ratings, Ad Fontes also assigns a score to news sources based on a weighted average of the pieces of content published by that news source. To validate our slant estimation approach, we compare our Twitter-based slant estimates with Ad Fontes scores at both video-level and channel-level.

For validating our slant estimates at the video-level, we sampled 300 videos from Ad Fontes, were able to collect tweets for 286 of them, and then estimated their slant using our Twitter-based approach. Validation at the video-level was then carried out by comparing the ranks of the videos based on our Twitter-based slant estimates with the scores obtained from manual labeling by Ad Fontes. Ideally, when ordered from *very-left* to *very-right* ideologies, the rank of the videos in Ad Fontes labeling should be the same as the rank of the videos in ours. The ranks obtained by both methods for the sampled channels can be seen in Figure S2. It is evident that the ranks follow closely along the diagonal ($x = y$), indicating a high correlation between the scores computed by the two approaches. We quantify this correlation by computing Spearman's rank correlation metric (17), which at $\rho = 0.79$ (p-value < 0.05) indicates that the ranks of the two lists ordered left-to-right strongly co-vary. This shows that our automated Twitter-based approach is highly correlated with an expert-based manual coding approach at the video-level.

To validate our slant estimates at the channel-level, we sampled 100 outlets from Ad Fontes, identified their corresponding YouTube channels, and computed a channel-level slant estimate by averaging the slant estimates from the most popular videos on that channel. The ranks obtained by both methods for the sampled channels can be seen in Figure S3. We again see that the ranks follow closely along the diagonal and that Spearman's rank correlation metric (17), which at $\rho = 0.88$ (p-value < 0.05) indicates that the ranks of the two lists ordered left-to-right strongly co-vary. This further shows that our Twitter-based slant estimation approach very well aligns with an expert-based manual coding approach at the channel-level as well.

To illustrate the importance of video-level slant estimation, as opposed to channel-level, Figure S4 plots the distribution of the slants estimated for the most popular videos from six well-known channels. Note that while the distributions for the most ideologically extreme channels (i.e., One America News Network (OANN) and Democracy Now!) are skewed and relatively concentrated, the distributions of ideologically moderate channels (e.g., The Hill and CNN) have a large spread. This suggests that simply assigning the overall slant of a YouTube channel to all of its videos can lead to inaccurate estimates. This limitation applies to several prior audits which, for example, labelled all videos from CNN as *left* (1, 5, 8). In contrast, our method clearly shows that not all videos from CNN are ideologically left. Thus, we conclude that channel-level slant estimation does not accurately represent the ideology of a wide range of videos posted by the channel.† In addition, our video-level estimation method allows us to automatically and at-scale classify the ideology of millions of individual videos, some of which may come

*https://adfontesmedia.com/interactive-media-bias-chart/

†Green et al. (18) had a similar finding for estimating the slant of newspaper articles versus news domains from which these articles come.

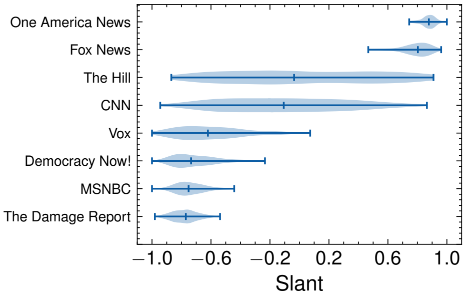

Fig. S4. Violin plot for the distribution of slant estimations for the top-100 most popular videos in the channel. The density of the plot at a given slant indicates the number of videos in the distribution around that point. The middle tick represents the mean of the distribution.

from channels that do not have ideological scores from existing work and that—as such—would not be reflected in parallel analyses.

In short, we are confident that the approach is robust and results in valid estimates. That said, we acknowledge that only the videos that have been shared on Twitter could have received a slant score and – as such – the unpopular videos that were not shared or had fewer than 12 landmarks would not be classified. This may lead to an under-classification of very niche videos (which would not receive a score and would not be included in our data). Yet, we find that this is not the case. Specifically, we examine the videos in our dataset from the list of problematic channels collected by (1) and (5) (more on this list in SM E) and find that the average coverage for these channels (i.e., the percentage of videos from these channels in our data for which we obtained a slant score) is 78.6%. Importantly, we also note that if the remaining 21.4% of videos were very extreme, we are underestimating our patterns, especially with regard to the results pertaining to radicalization.

C. Training Sock Puppets.

C.1. Source of training videos. The videos used to train the sock puppets were collected from 2,135 channels belonging to US-based journalists, politicians, media channels, and political pundits. The complete list of individuals and organizations was cataloged, shared with us, and later made public by Stefan McCabe, Jon Green and David Lazer [‡] by combining the following lists:

- Politicians
  - List of 747 politicians from the 116th and 117th members of Congress.
  - List of 51 state governors.
  - List of 617 political pundits collected by Green et al.
  - List of 788 politicians active during the COVID pandemic collected by Gallagher et al.
  - List of 6 recent presidential candidates.
- Media channels
  - A list of 77 media outlets collected by McCabe et al. through snowball sampling on tweeted news content from an initial list of well-known outlets. The initial seed list comprised outlets such as NYTimes, Fox News, Breitbart News, Washington Post, CNN, The Blaze, OANN, etc.

Of the cataloged list of 2,135 Twitter accounts, we identified 586 accounts which had dedicated YouTube channels. We did not consider Twitter handles that did not have a dedicated YouTube channel but rather had their videos posted on an organizational channel, e.g., videos of Tucker Carlson are posted on the Fox News YouTube channel but the two have dedicated Twitter handles. In cases like this, we only considered the organizational YouTube channel and discarded the individual account. We then collected at most the top 100 most watched videos from these channels, computed their slant using our Twitter approach, and categorized them into our five ideology categories used for training the sock puppets. Note that we did not consider the contents of the video itself rather the estimated slant so not all the videos used for training were necessarily political in nature. This approach enhances the ecological validity of our training and the resulting sock puppets, in that it is unlikely that even the most radical YouTube users only watch political videos.

C.2. External validation. One may argue that our approach to training sock puppets is unrealistic in the sense that real users are unlikely to have viewing patterns similar to our trained sock-puppets. To address this, we examine how similar our cases are to real user behavior by comparing the browsing histories of 2,462 adult Americans recruited via Lucid in 2019 (19). Every three months, the same participants completed 20-minute surveys and submitted - after extensive informed consent - their browsing data via our open-source tool that allows for transparent data sharing, Web Historian (20). From 31 million visits, we

[‡] https://github.com/sdmccabe/new-tweetscores

Table S1. Top-5 channels used for training sock puppets. The * indicates that these channels were also viewed by real users.

Very-left	Left	Center	Right	Very-right
MSNBC*	CNN*	ABC News*	ReasonTV*	Fox News*
Senator Bernie Sanders*	The Economist*	CBS News*	Fox News*	BlazeTV*
The Damage Report*	NBC News*	CNN	The Hill*	One America News*
Elizabeth Warren	The Atlantic*	NBC News*	Senator Marco Rubio	Newsmax
Vox*	Vox*	CNBC Television*	CBS News*	Senator Ted Cruz

identified 730,229 YouTube visits to 147,113 unique channels. We calculate the overlap between the channels watched by the participants and those from which we had videos for sock puppet training. This evidence suggests that the data used to train the sock puppets are also used by real people, with a 51.2% of channels used in data from humans also used in sock-puppet training. Specifically, from the 1,256 channels we used for training, 643 appeared in this dataset of real user watch histories. This overlap is substantial considering that (a) the majority of users do not regularly use YouTube for news consumption (21) and (b) the human subjects data were collected in 2019 and may not reflect the current state of channels that are popular on YouTube. The channels from which we most frequently source training videos appear in the real-user channel list and can be seen in Table S1. Note that some channels appear under two ideologies (e.g., Vox or CNN being in both *very-left*/*left* and Fox News being in both *very-right*/*right*) because we rely on video-level slant estimates and videos from these channels were generally classified under both ideologies.

D. Statistical Significance of Results. The statistic values and the significance of our results for the audit are in Table S2.

Table S2. Statistic values for the Kolmogorov-Smirnov test between the *center* and other sock puppet ideologies. Asterisks (*) indicate significant difference between *center* and the corresponding sock puppet ($p < 0.05$).

Ideology	Homepage	
	$n = 1$	$n = 8$
very-left	0.551*	0.261*
left	0.278*	0.140*
very-right	0.162*	0.180*
right	0.482*	0.362*

E. Problematic Channels. For the analysis involving problematic channels, we relied on the manual channel classifications by Ribeiro et al. (5) and Ledwich et al. (1), in similar fashion to Hosseinmardi et al (8). More specifically, we considered a video to be problematic if it belonged to a channel that was classified by Ribeiro et al. as either *intellectual dark web* (IDW), *Alt-lite*, or *Alt-right*. In the case of Ledwich et al.'s more granular classifications, we considered a video to be problematic if it belonged to a channel that was classified as one of *AntiSJW*, *Conspiracy*, *WhiteIdentitarian*, *Socialist*, *MRA*, *ReligiousConservative*, or *QAnon*. Thus, this analysis was carried out at a channel-level classification of either problematic or non-problematic. In total, we considered 4,150 channels as problematic, the breakdown of which can be seen in Table S3.

Considering the niche nature of problematic content, it was natural to assume that finding a decent signal in the tweets would be difficult as fewer people would have shared this content on Twitter (a pre-requisite for our video ideology slant estimation). However, as we mentioned in SM B.2, this was not the case in that we were able to estimate ideological slant for over 78.6% of the problematic videos in our dataset suggesting that even problematic content is frequently shared on Twitter.

Label	Ribeiro et al. (5)	Ledwich et al. (14)	Number of channels
IDW	✓		91
Alt-lite	✓		113
Alt-right	✓		88
AntiSJW		✓	1,056
Conspiracy		✓	2,413
MRA		✓	111
Socialist		✓	148
QAnon		✓	366
WhiteIdentitarian		✓	70
ReligiousConservative		✓	818

Table S3. Number of problematic channels in each label

F. Alternatively Trained Sock Puppets. In this section, we take a look at some alternative ways to train sock puppets and see what impact these trainings have on recommendations. Primarily, we are interested in sock puppets that are either trained on a single channel or on a truly heterogeneous diet of *very-left*, *center*, and *very-right* content.

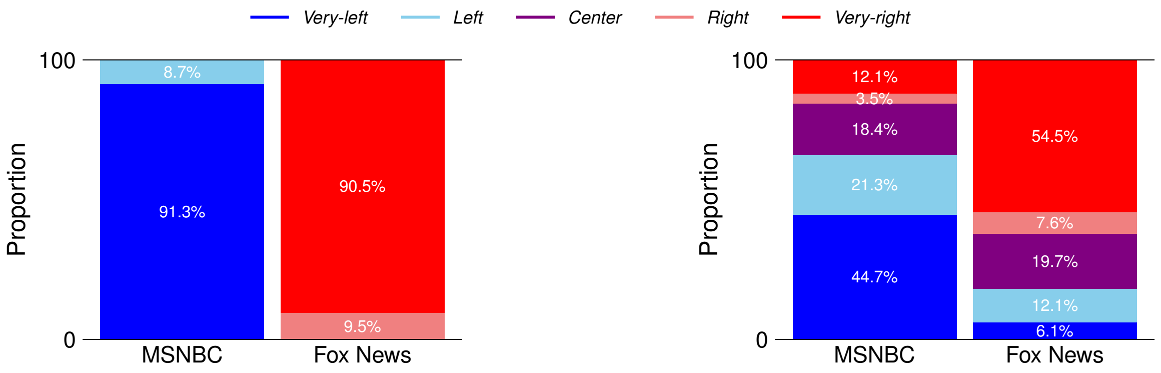

(a) $n = 1$ **(b)** $n = 8$

Fig. S5. The distribution of ideological categories in the top-n homepage recommendations for the MSNBC and Fox only sock puppets.

F.1. Single Channel Sock Puppets. For this analysis, we consider two news channels: MSNBC and Fox News. We train over thirty sock puppets for each channel and measure the congeniality of recommendations, as before. Figure S5 repeats the analysis of ideological congeniality in homepage recommendations for the single-channel sock puppets. We see in Figures S5a and S5b that there is ideological congeniality in the recommendations, i.e., the top-1 recommendation is almost 100% congenial to the ideology of the channel used in training (*very-left*/*left* for MSNBC and *very-right*/*right* for Fox News). With regards to which channel these recommendations come from: of the 184 homepage recommendations collected from the MSNBC sock puppets, only 23 were from MSNBC itself. Similarly, of the 176 recommendations collected from the Fox News sock puppet, only 21 were from Fox News itself. This indicates that only the ideologies of both MSNBC and Fox News were ingrained into the sock puppet's recommendations but not the channel itself. Furthermore, we see in Figure S6 that a growing proportion of these recommendations comes from problematic channels, indicating that potentially radical, conspiratorial, or "fringe" channels also appear in the recommendations despite the fact that the sock puppets were only trained on the more mainstream MSNBC and Fox News channels. Here, however, we observe that the left-leaning MSNBC-trained sock puppets are being recommended more problematic channels (up to 10%) than the right-leaning Fox News-trained sock puppets (up to 8%).

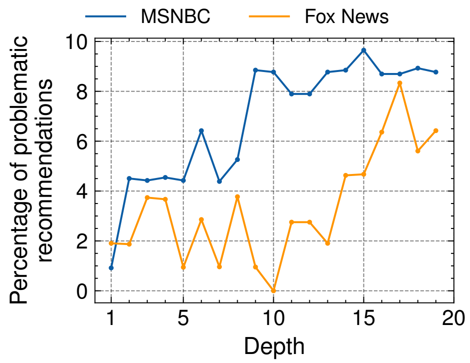

Fig. S6. Percentage of recommendations from problematic channels at each depth.

F.2. Heterogeneous Sock Puppets. We also test the recommendation congeniality and radicalization for truly ideologically heterogeneous sock puppets. We train over thirty sock puppets with the following constitution: 40% *very-left*, 20% *center* and 40% *very-right* training videos. Figures S7a and S7b show the ideological proportions for these mixed sock puppets at top-1 and top-8 homepage recommendations. We note that these recommendations are heavily right-slanted despite having a balanced composition of left and right content during training. This suggests that the recommendation system is more heavily influenced by *very-right* content. Furthermore, in Figure S8a the mixed sock puppet sees an increasing number of problematic recommendations in the up-next trail despite being trained on truly diverse videos (up to 20%).

G. Channel-level results. The results in the main paper use a video-level slant estimation approach. In this section, we revisit the results using a channel-level approach instead, where we take the mean of the slant of all the videos collected from a specific channel. Figure S11 repeats the result about congeniality in homepage recommendations seen earlier in the manuscript using channel-level slants instead. We observe similar results, albeit dampened, likely due to the earlier observation made in B.2 that not all videos from a channel share the same ideology. This aggregates more strongly slanted videos with less slanted

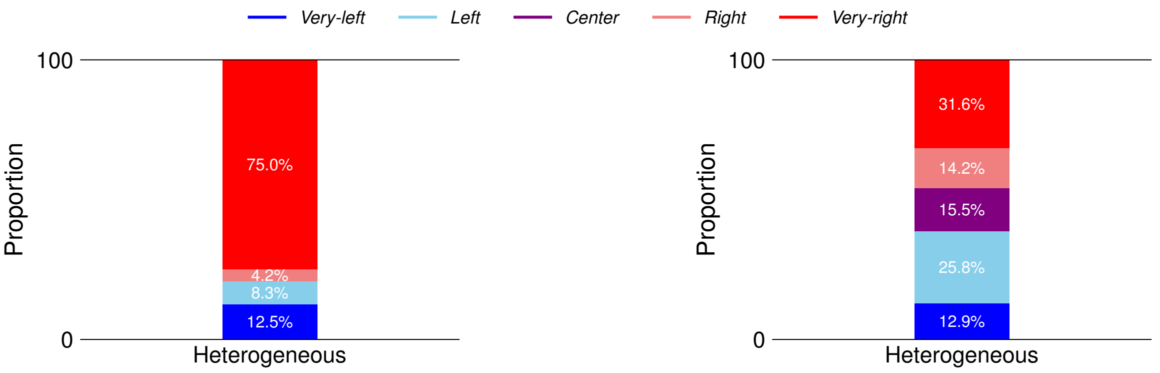

(a) $n = 1$

(b) $n = 8$

Fig. S7. The distribution of ideological categories in the top-n homepage recommendations for heterogeneous sock puppets.

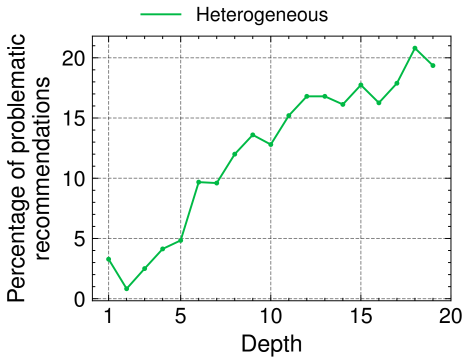

(a) Number of recommendations from problematic channels at each depth.

Fig. S8. Number of problematic recommendations in the up-next trail for the heterogeneous sock puppets.

videos from the same channel thereby reducing the overall slant at the channel-level. Also on figures S9a, S9b, and S10, we demonstrate the results for the exposure metric and slant radicalization.

References

1. M Ledwich, A Zaitsev, Algorithmic extremism: Examining youtube's rabbit hole of radicalization. *First Monday* **25** (2020).
2. K Roose, The Making of a YouTube Radical. The New York Times (https://www.nytimes.com/interactive/2019/06/08/technology/youtube-radical.html) (2019).
3. A Rodriguez, YouTube's recommendations drive 70% of what we watch. Quartz (https://qz.com/1178125/youtubes-recommendations-drive-70-of-what-we-watch) (2018).
4. E Pariser, *The Filter Bubble: What the Internet Is Hiding from You.* (Penguin Press), (2011).
5. MH Ribeiro, R Ottoni, R West, VA Almeida, W Meira Jr, Auditing Radicalization Pathways on YouTube in *ACM Conference on Fairness, Accountability, and Transparency (FAT*).* (2020).
6. M Chen, et al., Top-k off-policy correction for a reinforce recommender system in *ACM International Conference on Web Search and Data Mining.* (2019).
7. K Papadamou, et al., "It is just a flu": Assessing the Effect of Watch History on YouTube's Pseudoscientific Video Recommendations in *AAAI Conference on Web and Social Media.* Vol. 16, pp. 723–734 (2022).
8. H Hosseinmardi, et al., Examining the consumption of radical content on YouTube. *Proc. Natl. Acad. Sci.* **118** (2021).
9. AY Chen, B Nyhan, J Reifler, RE Robertson, C Wilson, Exposure to Alternative & Extremist Content on YouTube. Anti Defamation League (https://www.adl.org/resources/reports/exposure-to-alternative-extremist-content-on-youtube) (2021).
10. E Hussein, P Juneja, T Mitra, Measuring Misinformation in Video Search Platforms: An Audit Study on YouTube in *ACM Conference On Computer-Supported Cooperative Work And Social Computing (CSCW).* (2020).
11. K Papadamou, et al., "How over is it?" Understanding the Incel Community on YouTube in *ACM Conference On Computer-Supported Cooperative Work And Social Computing (CSCW).* (2020).

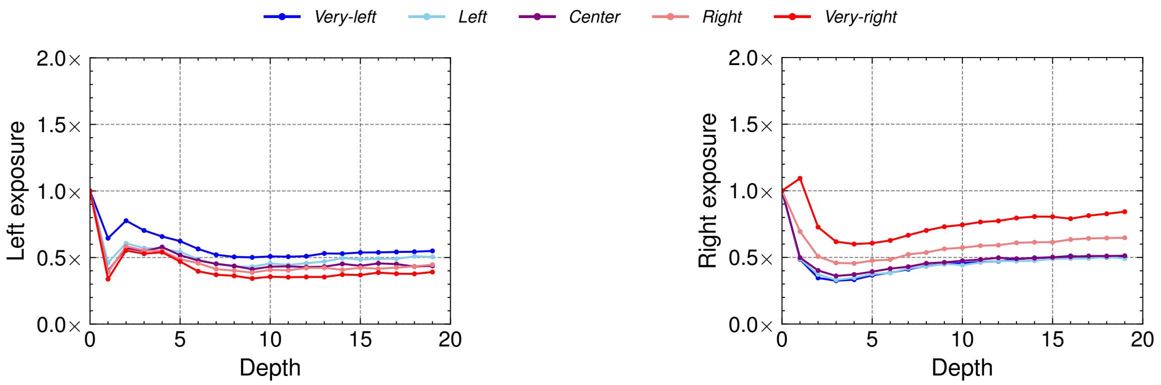

(a) Exposure to *very-left* videos

(b) Exposure to *very-right* videos

Fig. S9. Exposure to ideologically *very-left* and *very-right* videos at the **channel-level** in the up-next recommendations.

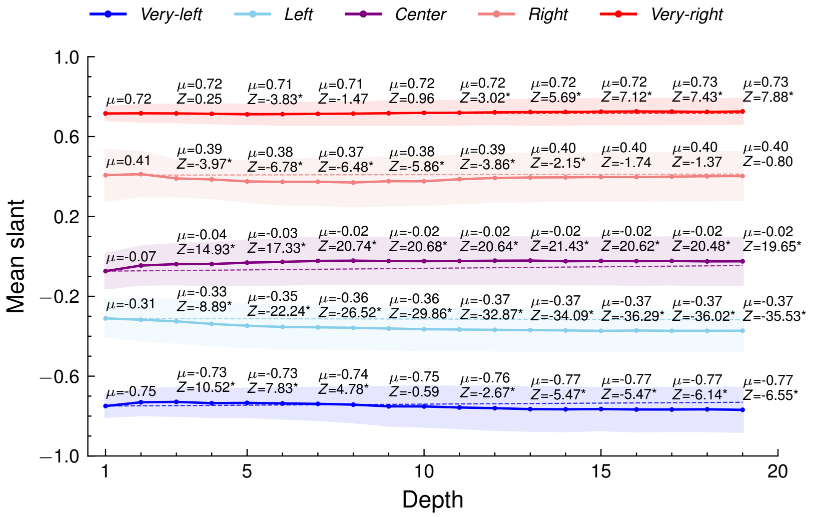

Fig. S10. The mean channel-level slant of the ideologically same videos watched by each sock puppet ideology in the up-next recommendations. For example, the top red line corresponds to the mean slant of the *very-right* videos watched by the *very-right* sock puppet.

12. L Sanna, R Salvatore, C Giulia, A Claudio, YTTREX: crowdsourced analysis of YouTube's recommender system during COVID-19 pandemic in *International Conference on Information Management and Big Data.* (2020).
13. M Faddoul, G Chaslot, H Farid, A longitudinal analysis of youtube's promotion of conspiracy videos (2020).
14. M Ledwich, A Zaitsev, A Laukemper, Radical bubbles on YouTube? Revisiting algorithmic extremism with personalised recommendations. *First Monday* **27** (2022).
15. H Le, Z Shafiq, P Srinivasan, Scalable News Slant Measurement Using Twitter in *AAAI Conference on Web and Social Media.* Vol. 11, (2017).
16. V Otero, Ad fontes media's first multi-analyst content analysis ratings project (2019).
17. Y Dodge, *Spearman Rank Correlation Coefficient.* (Springer New York, New York, NY), pp. 502–505 (2008).
18. J Green, et al., Curation bubbles: Domain versus url level analysis of partisan news sharing on social media (2021).
19. M Wojcieszak, et al., Null effects of news exposure: a test of the (un) desirable effects of a 'news vacation'and 'news binging'. *Humanit. Soc. Sci. Commun.* **9**, 1–10 (2022).
20. E Menchen-Trevino, Web historian: Enabling multi-method and independent research with real-world web browsing history data (year?).
21. M Walker, KE Matsa, News consumption across social media in 2021, Technical report (2021).

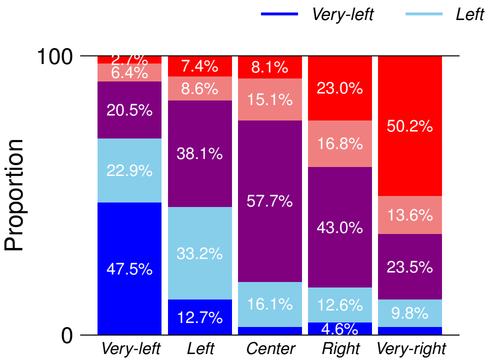

(a) $n = 1$

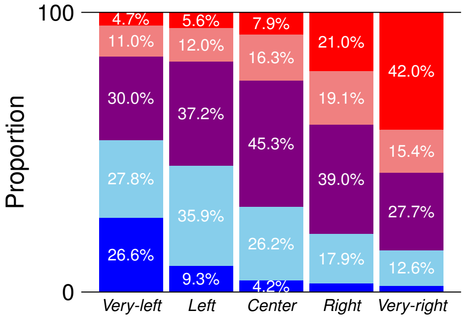

(b) $n = 8$

Fig. S11. The distribution of ideological categories in the top-n homepage recommendations at the **channel-level**. For the *very-left* sock puppet, the top-1 recommendation is 47.5% likely to be congenial.
